# Recent global outbreaks of highly pathogenic and low-pathogenicity avian influenza A virus infections

**DOI:** 10.1080/21505594.2024.2383478

**Published:** 2024-07-25

**Authors:** Hinh Ly

**Affiliations:** Department of Veterinary & Biomedical Sciences, College of Veterinary Medicine, University of Minnesota, Twin Cities, MN, USA

Depending on the host origin, influenza A viruses (IAVs) can generally be classified as those that can cause infections in humans or animals [1]. Avian IAVs normally circulate in wild and migratory birds but can occasionally cross species to infect humans, and hence, they are known as zoonotic avian influenza viruses (AIVs). Human cases of AIV infections can be acquired through either direct contact with the infected animal or through indirect contact with virus-contaminated materials (fomites) or environments. Zoonotic IAV infections, including those by AIVs, can result in mild symptoms of upper respiratory tract infection (cough, congestion, sore throat, sneezing) or ocular infection (conjunctivitis), but some cases of infection can progress to more severe forms of the disease (gastrointestinal and/or neurological symptoms, such as encephalitis and encephalopathy) that can be fatal. Avian IAVs include those that can cause a high degree of pathogenicity in humans upon infection. They are referred to as highly pathogenic avian influenza (HPAI) viruses and can include the influenza A(H5N1) and A(H7N9) viruses. In contrast, those avian IAVs that cause a low degree of pathogenicity are known as either low pathogenic avian influenza viruses or low-pathogenicity avian influenza (LPAI) viruses and include but are not necessarily limited to influenza A(H7N3), A(H9N2), and A(H10N3) viruses.

## Recent global outbreaks of human HPAI A(H5N1) virus infections

According to the US Centers for Disease Control and Prevention (CDC), a total of 912 sporadic HPAI H5N1 virus infections of humans have been reported since 1997 in 24 countries with a cumulative case fatality proportion of greater than 50% (https://www.cdc.gov/bird-flu/php/technical-report/h5n1–06052024.html). The number of human cases reached its peak in 2006 with 115 reported cases in 9 countries, and in 2015 with 145 reported cases in 4 countries that included a large outbreak of 136 human influenza A(H5N1) cases in Egypt. Besides four recent cases of zoonotic influenza A(H5N1) virus transmissions from dairy cattle to humans in the US (1 reported and confirmed case in Texas on 1 April 2024, 2 cases in Kansas on 22 May 2024 and 30 May 2024, and 1 case in Colorado on 3 July 2024) [2,3], most reported cases of human infections were associated with direct contacts with sick or dead poultry (that include but are not necessarily limited to 6 recently reported and confirmed human cases in Colorado on 14 July 2024 and a historical human case in Colorado reported on 28 April 2022) or visit to live poultry markets (LPMs) that could result in zoonotic virus transmissions. However, some rare and limited cases of human-to-human influenza A(H5N1) virus transmission have been reported in a small number of family members following prolonged and close exposure with a symptomatic patient [4,5]. The currently available epidemiological and virological evidence suggests that this virus has not acquired the ability to cause sustained transmission among humans. Thus, the likelihood of human-to-human transmission remains low.

Table 1 shows the reported human cases of global influenza A(H5N1) infections to the World Health Organization (WHO) from January 2022 to 4 June 2024 (https://www.who.int/westernpacific/wpro-emergencies/surveillance/avian-influenza). One of the most recent (and first) case of influenza A(H5N1) virus infection in Australia happened to a child, who had travelled to Kolkata, India, from 12 February 2024 to 19 February 2024 and returned to Australia on 1 March 2024. The family reported no known exposure to infected people or animals while in India. Upon their return to the US, however, the child felt ill and was hospitalized on 2 March 2024 and remained there for more than two weeks for treatment. Genetic sequencing of the patient’s sample identified the virus as the subtype influenza A(H5N1) virus and part of a strain that has been circulating in Southeast Asia and has been detected in previous human and poultry infections. Australia had experienced nine outbreaks of HPAI viruses in poultry farms since 1976. The most recent cases of avian IAV infection in poultry farms were reported in May 2024, when six farms near Melbourne were found to have the LPAI A(H7N3) virus strain circulating among poultry and the seventh farm was found to have the HPAI A(H7N9) virus strain among its flock.

**Table 1.** Reported global cases of human influenza A(H5N1) virus infections from January 2022 to June 4, 2024 (sources: WHO and CDC).

Country	Month of illness onset or case detection	Disease severity and outcome	Virus clade
Australia (after travel to India)	March 2024	Severe illness, survived	Clade 2.3.2.1a
Cambodia	February 2023	Critical illness, died	Clade 2.3.2.1c
	February 2023	Mild illness	Clade 2.3.2.1c
	October 2023	Critical illness, died	Clade 2.3.2.1c
	October 2023	Critical illness, died	Clade 2.3.2.1c
	November 2023	Critical illness, died	Clade 2.3.2.1c
	November 2023	Mild illness	Clade 2.3.2.1c
	January 2024	Severe illness, survived	Clade 2.3.2.1c
	January 2024	Severe illness, survived	Clade 2.3.2.1c
	January 2024	Critical illness, died	Clade 2.3.2.1c
	February 2024	Severe illness, survived	Not reported
	February 2024	Asymptomatic	Clade 2.3.2.1c
Chile	March 2023	Critical illness, survived	Clade 2.3.4.4b
China	September 2022	Critical illness, died	Clade 2.3.4.4b
	January 2023	Severe illness, outcome not reported	Clade 2.3.4.4b
Ecuador	December 2022	Critical illness, survived	Clade 2.3.4.4b
Spain	September 2022	Asymptomatic	Clade 2.3.4.4b
	October 2022	Asymptomatic	Clade 2.3.4.4b
United Kingdom	January 2022	Asymptomatic	Clade 2.3.4.4b
	May 2023	Asymptomatic	Clade 2.3.4.4b
	May 2023	Asymptomatic	Clade 2.3.4.4b
	July 2023	Asymptomatic	Clade 2.3.4.4b
	July 2023	Asymptomatic	Clade 2.3.4.4b
United States	April 2022	Mild illness (fatigue)	Clade 2.3.4.4b
	March 2024	Mild illness (conjunctivitis)	Clade 2.3.4.4b
	May 2024	Mild illness (conjunctivitis)	Clade 2.3.4.4b
	May 2024	Mild illness	Clade 2.3.4.4b
Vietnam	October 2022	Critical illness, survived	Not reported
	March 2024	Critical illness, died	Clade 2.3.2.1c

## Recent cases of human HPAI A(H7N9) virus infections

The influenza A(H7N9) virus had been detected in different bird species, including chickens worldwide. The virus was found in samples from the environment, including LPMs as well as some backyard poultry. The original low pathogenic A(H7N9) virus mutated into a form that was highly pathogenic for chickens [6]. According to the European Center for Infectious Disease Control and Prevention, most of the human HPAI A(H7N9) cases were noted between 2013 and 2017 with 28 reported human cases, and most of them were from Mainland China, including a few travel-related cases in patients who had visited Mainland China (https://www.ecdc.europa.eu/en/zoonotic-influenza/facts/faq-H7N9). According to the WHO, human infections with the influenza A(H7N9) virus were first reported in China in 2013. Specifically, on 31 March 2013, Chinese authorities reported identification of a novel zoonotic avian influenza A(H7N9) virus transmitted to humans that caused severe disease. However, since mass vaccination program against influenza A(H7N9) virus infections in poultry was implemented in China in September 2017, both the number of outbreaks and detections in poultry or environmental settings, as well as the number of human cases dropped significantly [7,8]. Since 2018, only very few sporadic human cases have been reported. This is a testament to the success and benefit of mass vaccination to prevent zoonotic virus transmissions.

## Recent cases of human LPAI A(H9N2) and A(H5N2) virus infections

Cases of human infection with LPAI viruses also appear to be on the rise in recent years. On 22 May 2024, the International Health Regulations (IHR) National Focal Point (NFP) for India reported to the WHO a case of human infection with the LPAI A(H9N2) virus. In that case, a 4-year-old child living in West Bengal state of India was tested positive for this virus (https://www.who.int/emergencies/disease-outbreak-news/item/2024-DON523). This is the second reported human infection of avian influenza A(H9N2) virus in India, with the first case occurred in 2019 in a 17-month-old boy, who was living in Melghat, India [9]. The infected infant had high intermittent grade fever, cough, breathlessness, and difficulty feeding for 2 days after illness onset. No history of the child’s exposure to any poultry was known at the time. The child received an antibacterial drug and antipyretics and fully recovered. This is an expected outcome for most human cases of LPAI virus infection.

However, the influenza A(H9N2) virus-infected child in West Bengal, who had a pre-existing medical condition with a previously diagnosed hyperreactive airway disease, suffered from a more severe form of the disease. He initially had a fever and abdominal pain (on 26 January 2024). A few days later, he developed seizures and was admitted to a paediatric intensive care unit (ICU) of a local hospital due to persistent and severe respiratory distress, recurrent high-grade fever and abdominal pain. The patient was diagnosed with post-infectious bronchiolitis caused by viral pneumonia. The patient tested positive for influenza B and adenovirus and was discharged from the hospital on 28 February 2024. However, the patient experienced another episode of severe respiratory distress and was again admitted to the paediatric ICU and intubated on 3 March 2024. A nasopharyngeal swab was collected and sent to the Kolkata Virus Research and Diagnostic Laboratory in India and was tested positive for influenza A (not sub-typed at the time) and rhinovirus. The same sample was also sent to the Indian National Influenza Centre at the National Institute of Virology in Pune for subtyping on 26 April 2024 and found to be influenza A(H9N2) virus via a real-time polymerase chain reaction (PCR). On 1 May 2024, the patient was discharged from the hospital. This patient had been exposed to poultry at home and in its surroundings. In both cases of human influenza A(H9N2) virus infections, the patients recovered fully.

Other recent human cases of influenza A(H9N2) virus infection have also been reported in Hong Kong, China, Bangladesh, and Pakistan [10–13]. According to the online report by the Hong Kong’s Centre for Health Protection (https://www.chp.gov.hk/files/pdf/2024_avian_influenza_report_vol20_wk23.pdf), there have been six reports of human A(H9N2) cases in mainland China that include two unrelated 3-year-old boys in Guangxi Zhuang Autonomous Region reported on 2 May 2024 and 2 February 2024, a 6-year-old boy in Anhui Province on 2 January 2024, an 11-year-old-boy in Jiangxi Province on 11 February 2024, another 3-year-old boy in Guangdong Province on 17 February 2024, and a 22-month-old girl in Hong Kong Special Administrative Region on 15 February 2024. Elsewhere in Vietnam’s Tien Giang Province, a 37-year-old man was found to be infected with influenza A(H9N2) virus on 10 March 2024; and a 59-year-old man in Mexico was found to be the first known case of avian influenza A(H5N2) virus infection. No other details were available about the medical conditions or how each of these persons likely contracted the virus.

## Recent cases of human LPAI A(H10N3) virus infections

Other LPAI viruses, such as the novel avian influenza A(H10N3) virus, have been frequently isolated in poultry in China in recent years [14,15] and have caused zoonotic infection in at least three confirmed human cases [16,17]. The first recorded human case of influenza A(H10N3) virus infection was a 41-year-old man with a medical history of hypertension and diabetes, who visited a live-poultry market (LPM) in Jiangsu Province, China, one week prior to becoming ill. The virus was isolated from the patient’s sample of bronchoalveolar lavage fluid at the time of admission on 22 April 2021 and sequenced confirmed to be a novel influenza A(H10N3) strain of Eurasian lineage and matched with influenza A(H10N3) sequences of three other specimens, including the duck’s drinking water, collected from the LPM where the man had visited, strongly implicating a zoonotic virus transmission event through contact with either an infected poultry or a contaminated environment at the LPM. A second human A(H10N3) case was of a 32-year-old man who had a medical history of fatty liver disease and was admitted to a hospital in Zhejiang Province, China on 18 June 2022 with a week of experiencing cough, haemoptysis, fever and diarrhoea. He raised chickens and ducks at home and worked in a sheep’s slaughterhouse and did not report contact with anyone with respiratory symptoms within the month of hospital admission. Next-generation sequencing of the patient’s sample of bronchoalveolar lavage fluid identified sequences matching other known influenza A(H10N3) virus sequences. The patient recovered fully after being treated with the antiviral Oseltamivir (brandname: Tamiflu) and discharged from the hospital.

According to the WHO, from 29 March 2024 to 3 May 2024, only one other new human case of influenza A(H10N3) virus infection was reported officially (https://www.who.int/publications/m/item/influenza-at-the-human-animal-interface-summary-and-assessment-3 May 2024). This case involved a 51-year-old man from Yunnan Province, China, with no underlying medical conditions. On 28 February 2024, the patient developed fever, cough, and shortness of breath that progressed to respiratory failure and was admitted to the hospital on 6 March 2024 with severe pneumonia. Testing of a sample collected from the patient on 15 March 2024 confirmed positivity for influenza A(H10N3) virus. The patient was working as a poultry and livestock farmer, who had been exposed to poultry and associated environment prior to symptom onset. At the time of reporting, the patient was admitted into the ICU for severe respiratory distress syndrome. This is the only known new (and third) case of human influenza A(H10N3) virus infection in China and globally to date.

## The One Health approach to monitor and control HPAI and LPAI virus infections

As the number of human cases of infection by HPAI or LPAI viruses increased globally in recent years, it is imperative that a coordinated and sustained effort of surveillance and reporting is maintained worldwide. It is arguably necessary to screen birds sold in LPMs for evidence of possible emergence of novel and/or more virulent strains of IAV and/or increased rates of morbidity and mortality in chickens. For instance, the author was involved in the original report of the full-length sequence of two chicken source influenza A(H7N9) viruses found in Guangdong’s LPM in China during a recent wave of human HPAI virus infections (from October 2016 to February 2017) [18]. The viruses under investigation were found to carry insertion of poly-basic amino acids (KGKRTAR/G) at the protease cleavage site of the viral haemagglutinin (HA) protein, which had previously been found in the HPAI (H7N9) virus strains in humans. Phylogenetic analysis of those novel avian IAVs suggested that their genomes reassorted between the Yangtze River Delta (YRD) and Pearl River Delta (PRD) virus clades. Molecular clock analysis indicated that they emerged several months before the emergence of the human HPAI H7N9 virus strains. Collectively, the finding suggested that influenza A(H7N9) viruses evolved in chickens through antigenic drift to include a highly pathogenic sequence signature in the HA gene (i.e. the poly-basic amino acids sequence motif) and highlight the need to perform routine sequence analysis as a risk assessment approach at the human-animal-environment interface. This type of a One Health approach requires both public and private supports to rapidly obtain and dispend knowledge about novel influenza A viral sequences and outbreaks to prevent potential future pandemics.
